# Dispositional Mindfulness and Psychological Health: a Systematic Review

**DOI:** 10.1007/s12671-017-0762-6

**Published:** 2017-07-01

**Authors:** Eve R. Tomlinson, Omar Yousaf, Axel D. Vittersø, Lauraine Jones

**Affiliations:** Department of Psychology, University of Bath, BA2 7AY, 10W 3.51, Bath, UK

**Keywords:** Mindfulness, Dispositional, Trait, Psychological health, Emotion, Cognition

## Abstract

Interest in the influence of dispositional mindfulness (DM) on psychological health has been gathering pace over recent years. Despite this, a systematic review of this topic has not been conducted. A systematic review can benefit the field by identifying the terminology and measures used by researchers and by highlighting methodological weaknesses and empirical gaps. We systematically reviewed non-interventional, quantitative papers on DM and psychological health in non-clinical samples published in English up to June 2016, following the Preferred Reporting Items for Systematic Reviews and Meta-analyses (PRISMA) guidelines. A literature search was conducted using PsycINFO, PubMED, Medline and Embase, and 93 papers met the inclusion criteria. Within these, three main themes emerged, depicting the relationship between DM and psychological health: (1) DM appears to be inversely related to psychopathological symptoms such as depressive symptoms, (2) DM is positively linked to adaptive cognitive processes such as less rumination and pain catastrophizing and (3) DM appears to be associated with better emotional processing and regulation. These themes informed the creation of a taxonomy. We conclude that research has consistently shown a positive relationship between DM and psychological health. Suggestions for future research and conceptual and methodological limitations within the field are discussed.

## Introduction

Mindfulness has been defined as the awareness that results from “paying attention in a particular way: on purpose, in the present moment, and non-judgmentally” (Kabat-Zinn 1994, p.4). Rooted in Buddhism, the concept of mindfulness has been drawing increasing interest within Western society. Mindfulness has been conceptualised and studied as both a state (i.e. a momentary condition) and a trait (i.e. a stable characteristic). State mindfulness can be enhanced by interventions such as mindfulness-based stress reduction and mindfulness-based cognitive therapy (Kabat-Zinn 1990; Segal et al. 2002). These interventions have been shown to positively influence psychological outcomes such as anxiety and mood disorders (Hofmann et al. 2010). The success of these interventions has sparked increased theoretical interest in the concept of mindfulness, leading to the exploration of mindfulness as an inherent human capacity or trait. Trait mindfulness, also known as dispositional mindfulness (DM) (Brown et al. 2007; Kabat-Zinn 1990), will be the focus of this review. DM has been found to occur at varying levels within the population, irrespective of mindfulness practice (Brown et al. 2007; Kabat-Zinn 1990). It has been found that regular mindfulness practice can lead to an increase in the baseline of the trait (Quaglia et al. 2016), indicating that mindfulness-based interventions also have the potential to deliver more than just short-term state changes.

In recent years, there has been an increase in research exploring the potential that DM may have in enhancing psychological health within the general population. So far, research into DM and health appears to echo that done with mindfulness interventions, with a previous review suggesting a range of benefits of DM on a variety of psychological health outcomes (Keng et al. 2011). For example, studies using non-clinical samples have shown an inverse association between DM and psychopathological symptoms such as depressive symptoms (Barnhofer et al. 2011; Bränström et al. 2011; Jimenez et al. 2010; Marks et al. 2010), post-traumatic stress disorder symptoms (Smith et al. 2011), borderline personality disorder symptomology (Fossati et al. 2011) and eating pathology (Adams et al. 2012; Lavender et al. 2011; Masuda et al. 2012). Furthermore, studies have shown significant negative associations between DM, stress (Brown et al. 2012) and anxiety (Hou et al. 2015) and significant positive associations between DM and psychological well-being (Bajaj et al. 2016a).

It is important to explore the relationship between DM and psychological health because it is likely to have implications for the individual’s self-management of health and well-being. With growing pressure on mental health services, there is an increasing need to promote a proactive approach to health self-management among the general population (Gilburt 2015). DM might be a resource that could be relied on in times of stress or symptomology to facilitate adaptive management of health and well-being (Bajaj et al. 2016a; Brown et al. 2012). It has been shown that DM can be enhanced through mindfulness meditation training (Quaglia et al. 2016). Therefore, if research suggests a positive link between DM and psychological health, more emphasis could be put on the promotion of mindfulness training as a psychosocial intervention for those with low DM. This could be useful not just with adults but also potentially within schools to enhance this adaptive trait within the younger generation. Accordingly, DM could be used as a baseline measure to shape patient-centred mindfulness interventions. DM is a multi-faceted construct, with facets including being able to observe and describe experiences, the ability to act with awareness and focus on the present and being able to be non-judgemental and non-reactive to experiences (Baer et al. 2006). It is likely that these facets will influence psychological health in different ways. Therefore, it is important to ascertain which facets are positive influences, as these can then be promoted within the population.

Despite the rapidly expanding research base exploring the relationship between DM and psychological health, a systematic review of these studies has not yet been conducted. A systematic review of this area is needed to provide a more integrated picture of the association between DM and psychological health. Such a review will benefit the field by informing the creation of a taxonomy. This will be useful to clearly show the areas of psychological health that have been studied in relation to DM, in turn aiding the identification of future research avenues. The review can also benefit the field by exploring the terms and measures used by researchers, which in turn will enable us to assess the consistency within the literature. Indeed, recent research has highlighted some issues related to DM measures and terminology, including a suggested over-reliance on measures assessing DM as a single construct, issues with factor structure of certain DM measures and a lack of distinction in papers between terms relating to DM and cultivated mindfulness (Rau and Williams 2016). Other measurement issues, such as a reliance on correlational analysis and violation of the assumptions of parametric tests through using ordinal data, may also affect the reliability and validity of DM research.

The aim of this paper is to systematically review quantitative empirical studies on dispositional mindfulness and psychological health in non-clinical samples, using the Preferred Reporting Items for Systematic Reviews and Meta-analysis (PRISMA) guidelines (Moher et al. 2009). The PRISMA guidelines, widely considered the best practice procedure, were followed to ensure the transparency and reliability of the review.

## Method

### Eligibility Criteria

#### Study Characteristics

Papers were included if they explored the relationship between DM and psychological health and did not involve interventions to manipulate mindfulness. This was because this review focused on DM, not on trained mindfulness. Experimental studies were included only if mindfulness was not part of the intervention. To decide if papers qualified as measuring an aspect of psychological health, the outcome measures used were appraised and the classification and specialisation of the journal the study was published in was also considered. For example, articles on pain were included only if the study explored a psychological aspect of the phenomenon, such as pain catastrophizing. Papers were included only if they used non-clinical samples. Non-clinical samples were selected because of the interest in DM and psychological health in the general healthy population. All studies in the review were quantitative, and they were included only if they used a validated measure of DM (e.g. the Mindful Attention Awareness Scale, Brown and Ryan 2003).

#### Report Characteristics

Papers were included if they were in English, empirical and peer-reviewed. Literature reviews and meta-analyses were also excluded. There were no restrictions on participant demographics such as age, sex, socio-economic status and year of publication.

### Search Strategy

The databases PsycINFO, PubMED, Medline and Embase were searched for papers published up until June 2016. Two search sets were used with the Boolean operators ‘OR’ and ‘AND’. The first search term related to the search terms disposition* OR trait. The second search term related to mindfulness and included the following search term ‘AND’ ‘mindful*’. The search terms entered were ‘Title’ in the ‘Fields’ search box and ‘All Years to Present’ in the Date ‘Published’ box’. Organic backward and forward searches were conducted to identify additional citations. Backward searches consisted of looking through the references of the identified papers for any other relevant articles. Forward searches were conducted by searching databases for relevant papers that had cited the already included articles.

### Quality Ratings

The papers included were subjected to quality rating using the Standard Quality Assessment Criteria for Evaluating Primary Research Papers from a Variety of Fields (The Alberta Heritage Foundation for Medical Research, February 2004). There are 14 criteria for quantitative studies that relate to the study design and rationale, sample size and characteristics and reporting of results. Each criterion, for example “Question/objective sufficiently described?” was assessed and awarded a score of ‘2’ if fulfilled, ‘1’ if partially fulfilled, ‘0’ if not present or unfulfilled and N/A if not applicable to the study. The maximum average score to be achieved is two. Two of the authors (ET and AV) first completed the quality ratings independently and then met to discuss their ratings and agree on final scores. Any discrepancies between raters were overcome through discussion and by revisiting the papers in question. These discrepancies were easily solved and agreed scores were saved.

#### Theme Identification

Two of the authors (ET and ADV) undertook a classification of the topics being studied in the literature and then arrived at the three main categories outlined in the emergent themes section of this paper and in the taxonomy. First, the authors began by determining and agreeing on the focus of the papers (e.g. depression, neuroticism and rumination) and then agreeing on their classification under meaningful categories. The topics of investigation were arranged under three umbrella categories, as it was found they fit easily under either cognitive, emotional or psychopathological aspects of psychological health, as discussed later. These umbrella categories, paired with the keywords taken from the papers as topics of investigation, then informed the creation of the taxonomy.

## Results

Ninety-three papers, all of which used quantitative methodology, met the eligibility criteria and were included in the systematic review (see Fig. 1 in supplemental data for an outline of the search process). The 93 papers studied a combined total of 34,620 participants. In total, 5287 was the largest study sample and 12 was the smallest. The research was based in a variety of countries, such as India, China, UK, USA and Ghana. Although the studies involved a range of ethnicities, the overall sample was primarily comprised of white Caucasian individuals.

**Fig. 1:**
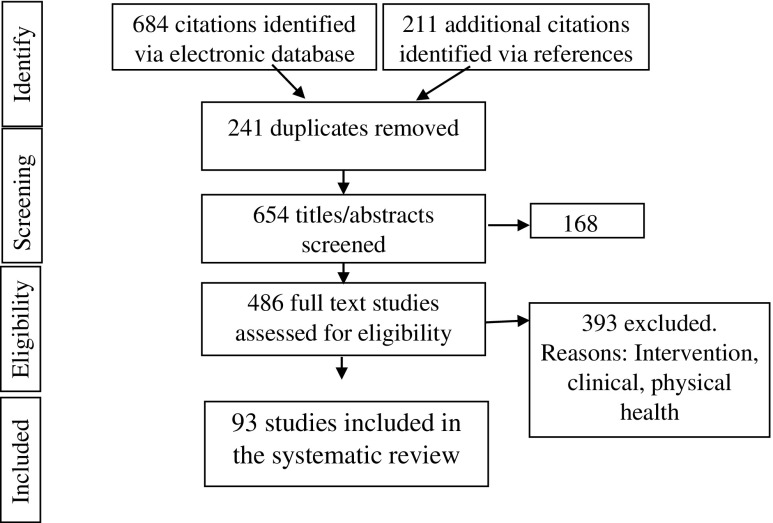
Search and inclusion/exclusion flowchart

Quality ratings for the 93 papers ranged from 1.55 to 2 (where below 1.6 was classified as low quality, 1.6–1.8 as medium and 1.8 and above as high). Five papers were deemed low quality, 29 papers as medium quality and 59 papers as high quality. This indicated a good standard of research in this area.

### Measures

Within the 93 papers, seven different instruments were used to measure DM. The most commonly used measure was the Mindful Attention Awareness Scale (MAAS; Brown and Ryan 2003), appearing in 48 papers. The MAAS measures mindfulness as a single construct. It consists of 15 items that detail an example of a lack of awareness and higher scores indicate greater mindfulness. It has been found to have adequate internal consistency (Cronbach’s alpha = .82; Baer et al. 2006). The second most widely used instrument was the Five Facet Mindfulness Questionnaire (FFMQ; Baer et al. 2006) used in 30 studies. This 39-item questionnaire measures five facets: acting with awareness, non-judging of inner experience, non-reactivity to inner experience, describing and observing. Each facet has high internal consistency (Cronbach’s alpha = .75 or above; Baer et al. 2006). Nine studies employed the Kentucky Inventory of Mindfulness Skills (KIMS; Baer et al. 2004). This 39-item questionnaire explores four subscales: observing, describing, awareness and accepting without judgment. This measure has been found to be reliable with good test-retest reliability. Test-retest correlations for the four subscales are: .65, .81, .86 and .83 respectively (Baer et al. 2004). One study used the extended version of this questionnaire, the KIMS-E, which consists of 46 items measuring the four subscales outlined above and also all seven items of the non-reactivity to inner experience factor from the FFMQ. One paper used the Freiburg Mindfulness Inventory (FMI; Walach et al. 2006), a 30-item scale with high internal consistency assessing mindful presence, non-judgmental acceptance, openness to experiences and insight (Cronbach’s alpha = .93; Walach et al. 2006). Two studies measured mindfulness using Cognitive and Affective Mindfulness Scale—Revised (CAMS-R; Feldman et al. 2007). This assesses four facets of mindfulness: attention regulation, awareness, non-judgmental acceptance and present-focus orientation. Finally, two studies assessed mindfulness skills by using the Children and Adolescent Mindfulness Measure (CAMM; Greco et al. 2011). Most papers used only one measure of mindfulness. Two papers used both the MAAS and FFMQ (Kadziolka et al. 2016; Woodruff et al. 2014), whilst one paper used the CAMS-R in conjunction with the FFMQ (Feldman et al. 2016). Test-retest reliability scores are lacking for most of these instruments (Park et al. 2013).

Non-DM measures were also used in the reviewed papers, as shown in Table 1. As there were so many of these, only a few of the most commonly used tools will be outlined here. The Depression, Anxiety and Stress Scale (DASS-21; Lovibond and Lovibond 1995) was frequently used within the papers. This 21-item self-report tool measures depression, anxiety and stress experienced over the last week on a 4-point Likert scale. The DASS-21 is a valid and reliable measure for use in non-clinical samples (Antony et al. 1998) with Cronbach’s alpha of .90, .84 and .84 for the depression, anxiety and stress subscales, respectively (Bhambhani and Cabral 2015). The Another Centre for Epidemiological Studies—Depression Scale (CES-D; Radloff 1977) was also frequently used to measure depressive symptomology. This is a 20-item Likert scale with good test-retest reliability (*r* = .057) and internal consistency (Cronbach’s alpha = .85–.90). Additionally, the Positive and Negative Affect Schedule (PANAS; Watson et al. 1988) was frequently used to measure affect. This scale requires participants to indicate how much they have experienced specific positive and negative emotions over the past few days by responding to words with a 4-point Likert scale. The positive and negative subscales are internally consistent (Cronbach’s alpha for negative affect = .084–0.87 and positive affect = .86–.90) with good test-retest reliability of *r* = .48 and .42 for positive and negative affect, respectively (Watson et al. 1988).

**Table 1:** Study characteristics of the included 93 articles on DM and psychological health

Authors	Measures	Methodology and analysis	*n*	Results	Psychological health factor	Quality rating
Adams et al. (2012)	FFMQ SSQ BULIT-R BSQ	Correlational; ANOVAs, chi-square analyses and hierarchical regression analyses	112. Students. Age: *M* = 20.00, SD *=* 1.69	HDM predicted lower bulimic symptoms	Eating disorder	1.82
Adams et al. (2014)	MAAS PANAS CES-D	Correlational; linear regression models	399. General. Age: *M* = 42.00, SD *=* 9.74	HDM predicted greater emotional stability during smoking cessation	Smoking	1.91
Adams et al. (2015)	MAAS HSI PHQ (3 scales)	Correlational; path analyses	399. General. Age: *M* = 42.00, SD *=* 9.74	HDM moderated lower stress and alcohol levels	Stress Alcohol	2.00
Alleva et al. (2014)	KIMS RRS QIDS	Correlational; mediation analysis	254. Students. Age: *M* = 21.40, SD *=* 2.30	Aspects of rumination (brooding, accepting without judgement, reflective pondering) mediate the link between mindfulness and depressive symptoms	Depressive symptoms	1.64
Bajaj et al. (2016a)	MAAS RSES PANAS SWEMWBS	Correlational; structural equation modelling	318. Students. Age: *M* = 20.30, SD *=* 1.30	Self-esteem (SE) fully mediated the link between DM, positive affect and mental well-being. SE also partially mediated the link between DM and negative affect	Well-being	1.80
Bajaj et al. (2016b)	MAAS RSES DASS	Correlational; structural equation modelling	417. Students. Age: *M* = 20.20, SD *=* 1.40	DM exerted indirect effect on anxiety and depression through SE	Anxiety Depression	1.80
Bakker and Moulding (2012)	MAAS HSPS AAQ-II DASS-21	Correlational; hierarchical regression analysis	111. General. Age: *M* = 31.07, SD *=* 11.95	HDM moderated SPS = lower levels of depression, anxiety and stress	Depression	1.73
Bhambhani and Cabral (2015)	CAMS-R DASS-21 NAS EQ	Correlational; mediation analyses	308. 69 general, age: *M* = 46.40, SD = 12.20, 239 students, age *M* = 22.30, SD = 7.00	DM and non-attachment are independent predictors of non-clinical psychological distress. These factors explain fully the effect of decentering on psychological distress.	Psychological distress	1.73
Bao et al. (2015)	MAAS WLEIS PPS	Correlational; multiple mediation model	380. General. Age: *M* = 27.21, SD = 5.10	DM = less stress	Stress	1.82
Barnes and Lynn (2010)	FFMQ BDI-II	Correlational; hierarchical linear modelling	102. Students. Age: *M* = 18.99, SD *=* 1.90	Acting with awareness, non-reactivity and non-judging inversely related to depressive symptoms. Observing directly related to depressive symptoms	Depressive symptoms	1.64
Barnhofer et al. (2011)	FFMQ EPQ BDI-II	Correlational; linear regression	144. General. Age: *M* = 43.00, SD *=* 6.80	HDM = low neuroticism/depressive symptoms	Neuroticism	2.00
Bergin and Pakenham (2016)	FFMQ LSPSS DASS SLS PWBS	Correlational; hierarchical multiple regression analyses	481. Students. Age: *M* = 21.90, SD *=* 5.78	DM = improved psychological adjustment (depression, anxiety, life satisfaction and dimensions of psychological well-being). DM important to mitigate effects of stress on depression and anxiety	Psychological adjustment	1.91
Bergomi et al. (2013)	FMI INC-S IAAM BSI PANAS	Correlational; structural equation modelling	376. General. Age: *M* = 40.40, SD *=* 18.40	DM moderates link between unavoidable distressing events and pathological symptoms/ negative affect	Pathological symptoms Negative affect	1.90
Bice et al. (2014)	MAAS Need Fulfilment Measure I-PANAS-SF CES-D	Correlational; linear regression analyses, mediation analysis	399. General. Age: *M* = 35.76, *SD =* 12.00.	DM positively associated with need fulfilment and both negatively associated with poor mental health outcomes (neg. Affect and depressive symptoms)	Negative affect Depressive symptoms	1.73
Black et al. (2012)	MAAS CES-D AQ PSS	Correlational; mediation path analysis	5287. Students. Age: *M* = 16.20, SD *=* 7.00	DM shields high pro-smoking intentions and low smoking refusal self-efficacy from turning into higher risk smoking behaviour	Smoking	2.00
Bluth and Blanton (2014)	CAMM PANAS SCS SLSS PSS	Correlational; bivariate correlations and mediation analysis	65. Students.	DM and self-compassion mediate pathway to emotional well-being	Emotional well-being	1.73
Bodenlos et al. (2015)	FFMQ PSS-14 SF-36 RAPI	Correlational; bivariate correlations and multiple hierarchical regression analyses	310. Students. Age: *M* = 19.70, SD *=* 1.30	DM observation facet negatively associated with physical health. Acting with awareness and non-judging positively linked to emotional well-being	Physical health Emotional well-being	1.82
Bowlin and Baer (2012)	FFMQ PWB SCS DASS	Correlational; ANOVA, chi-square and hierarchical regression analysis	280. Students. Age: *M* = 19.00	DM moderates between self-control and psychological symptoms	Depression	1.64
Bränström et al. (2011)	FFMQ HADS PSOM PSS	Correlational; ANOVA and multiple regression analyses	382. General	HDM diminishes stress and depression	Stress	2.00
Brown et al. (2012)	MAAS PSS POMS PANAS FNE Salivary Cortisol	Correlational; restricted maximum likelihood mixed models	44. Students. Age: *M* = 44.00, SD *=* 1.36	HDM lowers cortisol responses	Stress	1.67
Brown et al. (2015)	FFMQ SPWB SSRQ DTS CESD-R PSS PSWQ B-YAACQ	Correlational; structural equation modelling	994. Students	Distinct facets of DM relate to individual psychological health outcomes	Depressive symptoms Stress Anxiety Alcohol	1.82
Brown-Iannuzzi et al. (2014)	FFMQ PRS DES BDI	Correlational; multiple regression	624. General. Age: *M* = 40.93, SD = 9.60	DM dampens relationships between depressive symptoms related to discrimination	Depression	1.82
Bullis et al. (2014)	KIMS ASI SFS STAI-T Distress tolerance Heart rate SUDS STAI-B DSQ	Correlational; hierarchical regression model	48. General. Age: *M* = 29.10, SD = 8.32	DM reduces heart rate activity and anxiety during CO_2_ challenge -firemen	Stress	1.82
Christopher et al. (2013)	MAAS RAPI EIS ICSRLE	Correlational; hierarchical linear regression and mediational model	125. Students. Age: *M* = 24.00, SD *=* 8.00	Impulsivity mediated relationship between DM and alcohol-related problems	Alcohol use and problems	1.73
Ciesla et al. (2012)	MAAS PANAS-X RSQ Daily stress	Correlational; hierarchical linear regression	78. General. Age: *M* = 16.73, SD *=* 1.33	DM lowers levels of dysphoric mood in adolescents. DM = less rumination	Rumination	2.00
Coffey and Hartman (2008)	FFMQ TMMT TLI RRQ BSI	Correlational; structural equation modelling	258. Students. Two samples. Age: *M* = 18.90, SD *=* 1.20 and *M* = 18.75, SD = 1.20	DM lowers levels of dysphoric mood in adolescents	Stress	1.80
Cole et al. (2014)	MAAS ER89 STAI-Trait CES-D AESI	Correlational; hierarchical regression analyses	431. Students. Age: *M =* 22.40, SD = 3.20	DM buffered positive relationship between academic stress and depression but not anxiety	Academic Stress Psychological well-being	1.64
Daubenmier et al. (2014)	FFMQ STAI PSS RRQ PANAS Salivary cortisol	Correlational; regression analyses;	43. General	LDM = psychological distress and CAR	Stress	1.91
Day et al. (2015)	KIMS PCS PSWQ	Correlational; MANOVA	214. Students. Age: *M* = 18.70, SD *=* 2.30	PCS scores lower due to DM	Pain	1.80
de Frias (2014)	MAAS MMSE PHQ MCQ MOS ERQ	Correlational; hierarchical regression analyses	134. General. Age: *M* = 65.43, SD *=* 9.50	DM positively related to mental health. DM buffers negative effects of life stress on mental health	Mental health	1.82
Deng et al. (2014)	MAAS BDI SART	Correlational; Pearson’s correlation coefficient	23. Students. Age: *M* = 21.90, SD *=* 1.60	Depression negatively related to DM	Depression	1.27
Feldman et al. (2016)	Study 1: CAMS-R FFMQ PANAS Heart rate Skin conductance	Study 1: Correlational Hierarchical regression analyses	Study 1: 97. Students. Age: *M* = 20.48, SD = 4.12	Both studies found that higher DM = lower emotional reactivity to aversive experiences	Emotional reactivity	1.82
	Study 2: FFMQ PANAS BDEFS	Study 2: Correlational; multilevel modelling procedures	Study 2: 224. Students. Age: *M* = 19.71, SD = 3.02 (study 2).			
Feltman et al. (2009)	Study 1: MAAS Neuroticism scale Trait anger scale	Correlational; hierarchical regression	Study 1: 195. Students	DM moderates pernicious neuroticism	Neuroticism	1.55
	Study 2: MAAS, Neuroticism scale BDI	Correlational; hierarchical regression	Study 2: 94. Students			
Fetterman et al. (2010)	FFMQ Neuroticism scale Impulsivity scale	Correlational; regression analyses	226. Students	HDM = lower impulsivity; higher self-control and mediates neuroticism	Neuroticism	1.73
Fisak and Von Lehe (2012)	FFMQ PSWQ	Correlational; bivariate correlations and hierarchical regression analyses	400. Students. Age: *M* = 21.67, SD *=* 4.95	DM facets non-reactivity, non-judgment and acting with awareness, significantly predicted worry symptoms	Worry symptoms	1.73
Fogarty et al. (2015)	FFMQ Heart rate Physical activity status scale PANAS	Longitudinal; mixed-model ANCOVAs, MACOVA	80. General	DM = facilitates more adaptive emotional responding under stress	Emotional stress and differentiation	1.83
Fossati et al. (2011)	MAAS PDQ-4 BPD scale ASQ	Correlational; stepwise multiple regressions and mediation analysis	501. Students. Age: *M* = 17.22, SD *=* 0.88	DM mediates need for approval and BPD features	Borderline Personality Disorder	1.73
Gilbert and Christopher (2010)	MAAS CCI CES-D	Correlational; hierarchical linear regression analysis	278. Students. Age: *M* = 22.10, SD *=* 6.22	DM moderates depression	Depression	1.73
Gouveia et al. (2016)	MAAS IM-P SCS PSI-SF	Correlational; regression-based pth analyses	333. General. Age: *M* = 42.32, SD = 5.66	Higher DM & self-compassion associated with greater mindful parenting which is associated with lower parenting stress	Stress	1.91
Harrington et al. (2014)	KIMS SRIS PWB	Correlational; MANOVA	184. Students. Age: *M* = 19.70, SD *=* 1.33	DM positively correlated to psychological well being	Well being	1.64
Hertz et al. (2015)	FFMQ ECR Salivary cortisol PANAS VAS	Experimental; mediation models	228. General. Age: *M* = 21.31, SD = 6.12	DM associated with lower cortisol during conflict via attachment avoidance. DM predicted less negative affect and more positive cognitive appraisals post-conflict via lower attachment anxiety	Stress	1.80
Hou et al. (2015)	MAAS CAS-PA Salivary cortisol STAI PSS	Experimental; LCS modelling	105. Students. Age: *M* = 21.00, SD *=* 1.16	DM increases CAR and decreases anxiety	Anxiety	1.90
Howell et al. (2008)	MAAS Well-being scale SQS	Correlational; path analysis	305. Students. Age: *M* = 21.10, SD *=* 4.91	DM predicts sleep quality and well being	Well being	1.80
Howell et al. (2010)	MAAS SQS Glasgow sleep effort scale Pre-Sleep arousal scale Sleep hygiene index Epworth sleepiness scale Dysfunctional belief and attitudes scale	Correlational; structural equation modelling	334. Students. Age: *M* = 20.89, SD *=* 4.98	DM positively regulates sleep quality	Well being	1.80
Jacobs et al. (2016)	KIMS TEIQue-SF DASS-21 MHB	Correlational; path analyses	427. General. Age: *M* = 34.10, SD = 9.90	DM facets linked to multiple health behaviours	Stress Multiple health behaviours	1.90
Jimenez et al. (2010)	FMI CES-D NMR-15 mDES PWBS	Correlational; structural equation modelling	514. Students	DM lowers depression	Depression	1.90
Kadziolka et al. (2016)	FFMQ MAAS SCI Mindfulness practice – history questionnaire. ECG & heart rate Skin conductance	Experimental; bivariate correlations, ANOVAs	47. General. Age: *M* = 22.21, SD = 2.90	High DM associated with more effective down-regulation (parasympathetic nervous system activity, returning body to baseline) following stress	Stress	1.64
Kangasniemi et al. 2014)	KIMS Physical activity AAQ-2 SCL-90 BDI-II	Experimental. ANOVA and ANCOVA.	108. General. Age: *M* = 43.00, SD = 5.20	Higher DM = Higher self-reported physical activity and less psychological and depressive symptoms. Correlation also found between objectively measured physical activity and psychological well-being	Physical activity Depressive symptoms	1.91
Kiken and Shook (2012)	MAAS DAS LMSQ FES BDI-II BAI PANAS	Correlational; structural equation modelling	181. Students. Age: *M* = 19.40, SD *=* 3.40	DM reduces emotional disorders	Emotional distress	1.91
Kong et al. (2016)	MAAS PANAS SPWB rsFMRI	Experimental; correlational analysis, linear regression	290. Students. Age: *M* = 21.56, SD = 1.01	Individual differences in DM linked to spontaneous brain activity. DM engages brain mechanisms that differentially influence hedonic and eudaimonic well-being	Well-being	1.82
Lamis and Dvorak (2014)	MAAS NAS BDI-II SAEI-28 MCSD-B	Correlational; mediational model	552. Students. Age: *M* = 19.85, *SD* = 1.66	Depressive symptoms and suicide rumination negatively associated with DM and non-attachment. DM-suicide rumination association in part mediated by depressive symptoms	Depressive symptoms Suicide rumination	2.00
Lattimore et al. (2011)	Study 1: TFEQ-R21 KIMS HADS	Both studies: correlational; Pearson’s correlations	386 total. Study 1: students. Age: *M* = 21.00, SD *=* 5.50	DM reduces emotional eating in females	Eating disorder	1.91
	Study 2: FFMQ HADS TEFQ-R21 BIS-11		Study 2: Age: *M* = 26.00, SD *=* 0.60			
Laurent et al. (2013)	FFMQ CES-D Salivary cortisol	Experimental; dyadic growth curve modelling	100 couples. Age: *M* = 21.31, SD = 6.12	Women’s DM (non-reactivity facet) predicted higher conflict cortisol levels. Men’s DM (describing facet) predicted lower cortisol reactivity	Stress	1.91
Lavender et al. (2009)	MAAS BULIT-R WBSI	Correlational; hierarchical regression analyses	406. Students. Age: *M* = 19.10, SD *=* 1.50	HDM negatively associated with bulimic symptoms	Eating disorder	1.55
Lavender et al. (2011)	KIMS EAT-26 DASS-21	Correlational; hierarchical regression analyses	406. Students. Age: *M* = 19.10, SD *=* 1.50	HDM suggests lower levels of eating pathology among young adult women	Eating disorder	1.73
Mahoney et al. (2015)	MAAS KIMS ASI-3 AAQ-II BAI GAS STAI-Y1	Correlational; chi-square, independent *t* tests, Pearson’s correlations	511. Younger adults age: *M* = 20.10, SD = 2.50. Older adults age: *M* = 71.80, SD = 7.30	DM significantly inversely associated with anxiety sensitivity, experiential avoidance, trait and state anxiety	Anxiety	1.90
Malinowski and Lim (2015)	FFMQ UWES-9 WEMWBS PCQ JAWS	Correlational; structural equation modelling	299. General. Age: *M* = 40.10, SD = 11.60	DM predicts work engagement and well-being	Wellbeing	2.00
Marks et al. (2010)	MAAS IHSS-RLE RTSQ DASS-21	Correlational; multiple regression analyses	317. Students. Age: *M* = 16.10, *SD =* 1.10.	DM reduces depression, anxiety and stress due to life hassles	Stress	1.91
Masuda et al. (2010)	MAAS IRI-PD SCS	Correlational; multiple regression	625. Students. Age: *M* = 20.40, SD *=* 4.20	DM inversely related to psychological ill health and emotional distress	Emotional Distress	1.91
Masuda and Wendell (2010)	MAAS MAC-R GHQ-12 IRI-PD	Correlational; linear regression analyses	795. Students. Age: *M* = 20.40, SD *=* 4.20	DM mediates the relationship between disordered eating-related cognitions and psychological distress	Eating disorder	1.82
Masuda et al. (2012)	MAAS EAT-26 GHQ-12 MAC-R AAQ-16	Correlational; hierarchical multiple regressions	278. Students. Age: *M* = 20.88, SD *=* 4.30	DM moderates disordered eating	Eating disorder	1.91
McDonald et al. (2016)	MAAS DASS-21 DERS ECR-R	Correlational; *T* tests, chi-square, Pearson’s correlations	402. General	DM inversely related to distress, mediated by anxiety and emotion regulation deficits	Distress	2.00
Michalak et al. (2011)	KIMS RSE BDI	Correlational; hierarchical regression analyses	216. Students. Age: *M* = 24.80, SD = 7.60	Self-esteem more strongly associated with depression in LDM	Depression	1.64
Mun et al. (2014)	FFMQ PCP-S PCS CPAQ	Correlational; structural equation modelling	335. Students. Age: *M =* 19.62, SD = 3.00	DM mediates pain severity, catastrophising and impairment	Pain	2.00
Murphy and MacKillop (2012)	FFMQ AUDIT-C UPPS-P MCQ	Correlational; hierarchical regression analyses	116. Students. Age: *M* = 20.30, SD = 1.30	Effects of DM on alcohol consumption mediated by impulsivity	Alcohol	1.91
Ostafin et al. (2013)	FFMQ CPS IAT	Correlational; multiple regression analyses	61. Students. Age: *M* = 19.60, SD = 1.90	DM inversely related with alcohol preoccupation	Alcohol	1.73
Paolini et al. (2012)	MAAS CCEBstate FCQstate PFS	Experimental; Spearman rank order correlations	19. General	Brain study shows younger adults with HDM able to return to DMN; older adults low in DM continued to be pre-occupied with food	Eating disorders	1.69
Pearson et al. (2015a)	MAAS LET PSWQ BYAACQ	Correlational; structural equation modelling	1277. Students	DM inversely related to alcohol-related problems, anxiety and depressive symptoms	Alcohol /anxiety /depression	1.82
Pearson et al. (2015b)	FFMQ CESD-R PSWQ ALS DTS	Correlational; Lo-Mendall-Rubin adjusted likelihood ratio test	94. Students. Age: *M* = 20.60, SD = 4.40	HDM associated with adaptive emotional outcomes, LDM associated with depressive and anxiety symptoms, affective instability and distress intolerance	Depression /anxiety	1.77
Petrocchi and Ottaviani (2016)	FFMQ CES-D RRS	Longitudinal; multiple regression analysis	41. Students. Age: *M* = 24.40, SD = 2.80	DM prospectively predictive of lower depressive symptoms and rumination	Depression	1.91
Pidgeon et al. (2013)	MAAS DASS-21 TFEQ-EE GNKQ	Correlational; bivariate correlations, moderation analysis	157. General	DM is a moderator between psychological distress and engagement in emotional eating,	Eating disorder	1.73
Prakash et al. (2015)	MAAS PSS DERS WBSI	Experimental; bivariate correlations, simple mediation models	100. General	DM reduces stress	Stress	1.82
Prazak et al. (2012)	KIMS Heart rate SWBS WBI DS14	Correlational; multiple regressions	506. Students. Age: *M* = 21.40, SD = 4.80	HDM associated with better cardiovascular and psychological health	Cardiovascular /mental health	1.55
Raes and Williams (2010)	KIMS-E LARSS BDI-II MDQ	Correlational; hierarchical regression analyses	164. Students. Age: *M* = 19.21, SD *=* 0.91	DM reduces uncontrollable ruminative cycles	Depression	1.55
Raphiphatthana et al. (2016)	FFMQ BAI CES-D	Correlational; exploratory factor analysis	284. Students	DM facets predictive of anhedonia over time	Depression /mental health	1.70
Rasmussen and Pidgeon (2011)	MAAS RSES SIAS	Correlational; mediation analysis	205. Students. Age: *M* = 23.10, SD = 6.70	DM predictive of high self-esteem and low levels of social anxiety	Anxiety	1.64
Richards et al. (2010)	MAAS Self care scale SRIS SOS-10	Correlational; mediation analysis	148. General. Age: *M* = 42.30, SD = 14.90	DM mediates the relationship between self-care and well-being	Well-being	1.73
Short et al. (2016)	FFMQ PANAS DASS-21 SCMS BRIEF PRF-IN DKEFS	Correlational; correlational analysis, multiple mediator models	77. Students. Age: *M* = 21.20, SD = 6.00	Executive functioning and self-regulation mediates the inverse relationship between DM and negative affect	Well-being	1.82
Sirois and Tosti (2012)	MAAS GPS PCS SF-36	Correlational; structural equation modelling	339. Students. Age: *M* = 21.70, SD *=* 4.90	DM mediates procrastination and stress	Stress	1.80
Slonim et al. (2015)	FFMQ HPLP-II DASS	Correlational; canonical correlation and MANOVA	207. Students. Age: *M* = 21.80, SD = 3.60	DM associated with distress and self-care	Distress /well-being	1.55
Smith et al. (2011)	MAAS AUDIT BDI-II Firefighter stress LOT-R PMS PHQ-15 PDS ISEL	Correlational; hierarchical multiple regression analyses	124. General. Age: *M* = 33.70, SD *=* 8.13	MD = fewer PTSD symptoms	PTSD	1.73
Soysa and Wilcomb (2015)	FFMQ SCS-Short Self-efficacy scale DASS-21 WEMWBS	Correlational; hierarchical regression analyses	204. Students	DM predictive of stress, depression, anxiety and well-being	Stress /depression /anxiety /well-being	1.82
Tan and Martin (2016)	CAMM DASS-21 RSES RSCA AFQ-Y8	Correlational; regression analyses	106. General. Age: *M* = 15.00, SD = 1.20	DM negatively associated with stress, anxiety, depression, cognitive inflexibility, and a positive association with self-esteem and resiliency	Stress /depression /anxiety /well-being	1.91
Vinci et al. (2016)	FFMQ DMQ-R AUDIT	Correlational; linear regression analyses	207. Students. Age: *M* = 20.10, SD = 1.90	Coping motives and conformity motives mediate the relationship between DM and problematic alcohol use	Alcohol	1.82
Vujanovic et al. (2007)	MAAS ASI MASQ ASQ BVS	Correlational; hierarchical multiple regression analyses.	248. General. Age: *M* = 22.40, SD = 7.90	DM with anxiety sensitivity predictive of anxious arousal symptoms and agoraphobic cognitions	Anxiety	1.82
Walsh et al. (2009)	MAAS ECR-R NEO-PI-R	Correlational; regression analyses	153. Students. Age: *M* = 25.90, SD = 6.70	DM predicted by trait anxiety, attachment anxiety and attentional control	Anxiety	1.73
Wang and Kong (2014)	MAAS WLEIS GHQ-12 SWLS	Correlational; structural equation modelling	321. Students. Age: *M* = 27.20, SD = 5.40	Emotional intelligence partially mediates the effect of DM on distress	Distress	1.80
Waszczuk et al. (2015)	MAAS Mood and feelings scale CASI	Correlational; structural equation modelling	2118. Twins. Age: *M* = 16.30, SD = 0.70	DM is 33% hereditable and 66% due to non-shared environment, attentional control links DM to anxiety and depression sensitivity	Depression /anxiety	2.00
Weinstein et al. (2009)	MAAS Stress appraisal single item COPE Anxiety measure LOT	Correlational; hierarchical regression analyses	368. Students	DM = less use of avoidant coping strategies	Stress	1.82
Wenzel et al. (2015)	KIMS WHO-5 BFI	Correlational; hierarchical linear regression	1147. General. Age: *M* = 34.30, SD = 11.90	DM mediator for high levels of neuroticism	Neuroticism	1.82
Woodruff et al. (2014)	MAAS FFMQ SCS AAQ-II BAI BDI-SF SWLS QOL-BREF PANAS	Correlational; regressions	147. Students	DM predictive of psychological health, but non-significant when self-compassion and psychological inflexibility are considered	Psychological health	1.64
Wupperman et al. (2008)	MAAS MEPS-Int MEPS-Emo PAI-BOR EPQR-A	Correlational; hierarchical regression analyses and structural equation modelling	342. Students	DM predicts BPD features	BPD	1.89
Zimmaro et al. (2016)	MAAS PSS Salivary cortisol PWB	Correlational; regression analyses	85. Students. Age: *M* = 19.34, SD = 1.35	HDM associated with lower perceived stress and cortisol, and greater psychological well-being	Stress /well-being	1.82

### Emergent Themes

Three main themes emerged when looking at the 93 papers. Thirty-nine studies focused on exploring the links between DM and *psychopathological symptoms*, such as symptoms of depression. Twenty-one studies investigated the *cognitive processes* that mediate the relationship between DM and psychological health, such as rumination. Forty-two studies explored *emotional factors*, such as emotional regulation, that were associated with DM. There was some overlap between studies as papers tended to use more than one outcome measure, e.g. depression and stress. Papers have been categorised as accurately as possible to their corresponding overarching theme; however, some appear twice due to focusing on more than one theme. The emergent themes informed the creation of a taxonomy, shown in supplemental data Fig. 2. The research comprising the three themes will be discussed below.

**Fig. 2:**
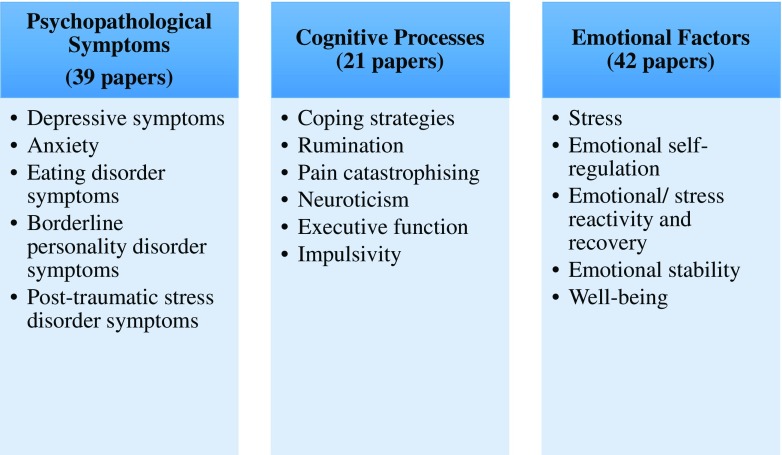
Taxonomy of the associations between DM and psychological health

#### Psychopathological Symptoms

Thirty-nine papers investigated the relationship between DM and psychopathological symptoms in non-clinical populations. The most commonly researched topic within these papers was the link between DM and depressive symptoms. Twenty-nine papers used depressive symptoms as an outcome measure; however, some of these will be covered under ‘cognitive processes’ as they focused mainly on cognitive mediating factors influencing the relationship between DM and depressive symptoms. A total of 21 papers focused on depressive symptoms (Bajaj et al. 2016b; Bakker and Moulding 2012; Barnes and Lynn 2010; Bergin and Pakenham 2016; Bice et al. 2014; Brown et al., 2015; Brown-Iannuzzi et al. 2014; Bhambhani and Cabral 2015; Deng et al. 2014; Gilbert and Christopher 2010; Jimenez et al. 2010; Kangasniemi et al. 2014; Marks et al. 2010; Michalak et al. 2011; Pearson et al. 2015a; Pearson et al. 2015b; Raphiphatthana et al. 2016; Soysa and Wilcomb 2015; Tan and Martin 2016; Waszczuk et al. 2015; Woodruff et al. 2014). All of these studies found a negative relationship between DM and depressive symptoms. Of particular interest, it has been suggested that DM may work to protect against the development of depression and other pathological symptoms (Gilbert and Christopher 2010) by buffering against negative factors such as discrimination (Brown-Iannuzzi et al. 2014), unavoidable distressing experiences (Bergomi et al. 2013), low self-esteem (Michalak et al. 2011), life hassles (Marks et al. 2010) and perceived stress (Bergin and Pakenham 2016). Most of these studies used samples of university students. Only one study out of these explored the links between DM and depressive symptoms in younger participants aged 13–18, also finding that DM is negatively associated with depression (Tan and Martin 2016).

It is well known that anxiety and depressive symptoms tend to co-occur in individuals. It is therefore not surprising that we found that nine of the papers exploring depressive symptoms also looked at anxiety as an outcome measure (e.g. Bajaj et al. 2016b; Bakker and Moulding 2012; Bergin and Pakenham 2016; Brown et al., 2015; Bhambhani and Cabral 2015; Marks et al. 2010; Pearson et al. 2015a; Pearson et al. 2015b; Soysa and Wilcomb 2015; Tan and Martin 2016; Waszczuk et al. 2015). As above, these papers found that DM was inversely related to anxiety. A further seven studies explored the relationship between DM and anxiety without measuring depressive symptoms. These studies further supported the beneficial influence of DM, finding that DM was negatively associated with anxiety sensitivity, trait and state anxiety and social anxiety (Fisak and Von Lehe 2012; Hou et al. 2015; Mahoney et al. 2015; Rasmussen and Pidgeon 2011; Vujanovic et al. 2007; Walsh et al. 2009).

Eating pathology and risk factors for disordered eating were explored in eight papers (Adams et al. 2012; Lattimore et al. 2011; Lavender et al. 2009; Lavender et al. 2011; Masuda and Wendell 2010; Masuda et al. 2012; Paolini et al. 2012; Pidgeon et al. 2013). Overall, it appeared that DM is negatively associated to eating pathology. For example, Lavender et al. (2009) found a negative association between DM and bulimic symptoms in a large sample of undergraduate men and women.

Despite not occurring as often as the abovementioned disorders, symptoms of Borderline Personality disorder (BPD) were explored in relation to DM in two papers (Fossati et al. 2011; Wupperman et al. 2008). Both papers found that DM was negatively associated with the number of BPD features, concluding that deficits in mindfulness may go some way to explain BPD features. Additionally, post-traumatic stress disorder (PTSD) was covered by one paper (Smith et al. 2011), finding that DM was associated with fewer PTSD symptoms in a sample of urban fire fighters.

Overall, papers exploring the link between DM and psychopathological symptoms are bolstered by using validated measures of DM (e.g. the MAAS) and reliable outcome measures (e.g. DASS-21). The studies predominantly use cross-sectional designs with suitable sample sizes for the methods of correlational analysis used. However, arguably the literature is limited due to participants’ ordinal responses, obtained through the employment of Likert style questionnaires, being analysed with parametric tests. It has been argued this violates the assumptions of parametric analysis (Field 2013). This should therefore be considered when reviewing the findings of the literature, as it may reduce the reliability and validity of the results.

#### Cognitive Processes

Twenty-one papers aimed to unravel the potential mediators of the influence of DM on psychological health. Most of these papers focused on how DM relates to cognitive thinking styles and how these styles impact on psychological health. For example, Kiken and Shook (2012) have found that, generally, individuals with higher DM are less likely to get caught up in negative cognitive thinking processes that are likely to leave them at risk of emotional disorders. Studies have suggested that DM is inversely associated with the use of avoidant coping strategies when in stressful situations (Weinstein et al. 2009; Sirois and Tosti 2012). An example of an avoidant coping strategy is procrastination, which has been found by Sirois and Tosti (2012) to be positively associated with poor health and negatively associated with DM. They found that DM mediates the effects of procrastination on health.

Rumination is another example of an avoidant coping strategy and a cognitive process that appears to have been researched frequently in relation to DM. Defined as repetitive thinking about a situation or mood and its consequences (Nolen-Hoeksema 1991), six papers in this review have focused on rumination (Alleva et al. 2014; Ciesla et al. 2012; Coffey and Hartman 2008; Petrocchi and Ottaviani 2016; Raes and Williams 2010; Lamis and Dvorak 2014). These studies have found that DM predicts reduced uncontrollable ruminative cycles and less suicidal rumination (Petrocchi and Ottaviani 2016; Raes and Williams 2010; Lamis and Dvorak 2014; Ciesla et al. 2012). Furthermore, two papers have found that DM is inversely related to pain catastrophizing, which is the tendency to ruminate on feelings of pain and experience increased helplessness (Day et al. 2015; Mun et al. 2014). Rumination is a risk factor for depression and psychological distress, and two studies have found that rumination does mediate the link between DM and depressive symptoms (Alleva et al. 2014) and psychological distress (Coffey and Hartman 2008). This suggests that DM might reduce rumination, which in turn protects against psychological ill health. In a similar vein, studies have indicated that DM is associated with reduced neuroticism, which is a trait that encapsulates negative thinking and is a risk factor for ill health (Barnhofer et al. 2011; Feltman et al. 2009; Wenzel et al. 2015).

One paper, by Short et al. (2016), aimed to find out how DM links to executive functioning. Results indicated that the ‘acting with awareness’ and ‘non-judgement of inner experience’ facets of mindfulness positively correlated with total executive function in a sample of students. The authors argue that individuals high in these traits are aware of changes internally and externally, which activate executive functions, allowing them to successfully navigate situations.

There appears to also be a literature exploring cognitive mediating factors between DM and addictive behaviours, such as smoking and alcohol use. A study by Black et al. (2012) has shown that DM helps to prevent smoking by buffering pro-smoking intentions and enhancing smoking refusal, whilst Ostafin et al. (2013)found that DM is inversely related to preoccupation with alcohol. Three papers have found that the relationship between DM and alcohol problems can be explained partly by personality traits: impulsivity and neuroticism (Christopher et al. 2013; Fetterman et al. 2010; Murphy and MacKillop 2012). Finally, one paper has found that lower coping motives in students mediate the link between mindfulness facets and alcohol use (Vinci et al. 2016).

Most of the papers exploring the relationship between DM and cognitive processes use cross-sectional designs featuring self-report measures which can be prone to response bias, therefore reducing the reliability of the results somewhat. However, it is worth highlighting that one study by Petrocchi and Ottaviani (2016)detailed a longitudinal exploration into DM, rumination and depressive symptoms. The researchers found that DM (specifically the facet ‘non-judge’) at time one had a protective function against depressive symptoms and rumination at time two (2 years later). Similar longitudinal studies are needed to form a reliable picture of how DM and psychological health interact over time. Petrocchi and Ottaviani’s (2016) study also indicated that four out of five of the FFMQ subscales (not ‘observe’) had high test-retest reliability. This is an interesting finding, suggesting that the psychometric properties of the FFMQ may not be that robust, which may have implications for the reliability of the results of the many studies in this area using the FFMQ.

#### Emotional Factors

Forty-two papers explored the link between DM and emotional factors. There is a large literature exploring the effects of DM on perceived stress, with 27 papers focusing on stress in this review. Overall, these studies have found that higher DM is associated with lower perceived stress (e.g. Bhambhani and Cabral 2015; Gouveia et al. 2016; Jacobs et al. 2016; Marks et al. 2010; Soysa and Wilcomb 2015; Tan and Martin 2016; Zimmaro et al. 2016) and emotional distress (Masuda et al. 2010). Studies suggest that DM buffers against the negative influence of perceived stress on psychological health (Adams et al. 2015; Bergin and Pakenham 2016; Bränström et al. 2011; Cole et al. 2014; Daubenmier et al. 2014). It appears that one of the possible mechanisms through which DM does this is by improving emotional regulation (Coffey and Hartman 2008; de Frias 2014; Feldman et al. 2016; Kadziolka et al. 2016; McDonald et al. 2016; Prakash et al. 2015). Individuals with higher DM have also been found to have lower emotional and stress reactivity to aversive situations and appear able to respond more adaptively when stressed (Brown et al. 2012; Bullis et al. 2014; Hertz et al. 2015; Laurent et al. 2013).

One recent study concluded that mindfulness reduces psychological stress by improving self-care, defined by the authors as behaviours that maintain or improve well-being (Slonim et al. 2015). Meanwhile, two papers suggest that emotional intelligence mediates the impact of mindfulness on mental distress and perceived stress (Wang and Kong 2014; Bao et al. 2015). Studies also suggest that that DM is linked to greater emotional stability during smoking cessation (Adams et al. 2014) and greater emotional differentiation (Fogarty et al. 2015).

In addition to stress, one other key emotional factor that emerged from this review to be associated strongly with DM is psychological well-being. The relationship between emotional well-being and DM has been developing interest within the field of positive psychology. In line with this, 13 papers in the present review were devoted to exploring this relationship (Bajaj et al. 2016a; Bluth and Blanton 2014; Bodenlos et al. 2015; Bowlin and Baer 2012; Harrington et al. 2014; Howell et al. 2008; Howell et al. 2010; Kong et al. 2016; Malinowski and Lim 2015; Prazak et al. 2012; Richards et al. 2010; Short et al. 2016; Zimmaro et al. 2016). All 13 papers demonstrated positive associations between DM and psychological well-being. Two papers stated more specifically that two facets of mindfulness ‘acting with awareness’ and ‘non-judgement’ were positively related to well-being (Bodenlos et al. 2015; Short et al. 2016). Although the majority of this research is self-report data, one study used resting-state functional magnetic resonance imaging (rs-fMRI) to show that DM engages specific brain that also influence hedonic (positive/negative affect) and eudaimonic (meaningful/purposeful life) well-being. This research furthers the field by demonstrating potential neurobiological mechanisms that influence well-being through DM (Kong et al. 2016).

Overall, studies exploring the emotional factors impacted by DM appear to suggest that DM is associated with a variety of adaptive emotional outcomes (Pearson et al. 2015b) such as emotional regulation, lower emotional and stress reactivity and improved recovery following a stressful situation. These are all factors that positively impact upon psychological health.

These studies have enlisted suitable sample sizes for the statistical analyses used, boosting the validity of the findings. However, almost all the papers are limited by the nature of the samples used. Over-reliance on the use of Western student samples, particularly Psychology undergraduates, reduces the external validity of the findings of many of these papers (e.g. Bluth and Blanton 2014; Marks et al. 2010). Additionally, sampling biased towards females (e.g. Howell et al. 2008) is also of concern. Few of these papers detail data screening or examination of distribution, making it hard to evaluate the suitability of the data for the statistical tests used. However, the few that do (e.g. Tan and Martin 2016) have normally distributed data with assumptions being met for statistical analysis.

## Discussion

This review has presented an integrated overview of the research exploring the links between DM and psychological health. The research explored a range of outcome measures, which we propose belong to three dominant themes (see supplemental data Fig. 2). Overall, DM appears to be positively associated with psychological health. The 93 included papers were generally deemed to be of a high research standard when assessed using the quality assessment criteria. Specific methodological limitations within the literature will be covered within this discussion.

Several meaningful results have been found, but perhaps one of the most prominent is the inverse relationship between DM and negative cognitive patterns. It appears that cognitive processes are a key mechanism through which DM affects psychological health. For example, rumination is a risk factor for psychological distress and depression (Nolen-Hoeksema et al. 2008), and studies suggest DM protects against rumination (Petrocchi and Ottaviani 2016). It is thought this is due to individuals high in DM having greater awareness but less attachment and judgement of thoughts (Brown et al. 2007). This reduces the repetitive focus and attenuation of thoughts that can lead to psychological distress and depression. Related to rumination, research has also demonstrated an inverse association between DM and pain catastrophizing (Day et al. 2015). Pain catastrophizing involves negative evaluation and emotional sensitivity, whereas DM involves non-judgmental acceptance. It appears that DM can enhance patient resilience and buffer against the development of negative thinking patterns that predict psychological ill health. This is a noteworthy finding that has implications at individual and societal levels. Proactive attempts to increase DM are likely to improve psychological well-being and equip individuals with healthy cognitive processes and emotional regulatory strategies. This will allow healthy individuals to remain resilient and present in the potential midst of diagnoses and long-term illness. Furthermore, as research suggests that DM is linked to the selection of adaptive stress-coping techniques (Weinstein et al. 2009), interventions to increase DM in non-clinical samples might reduce the somatisation of stress and potentially lessen the use of unhealthy coping strategies such as smoking, drinking and over-eating.

### Conceptual/Methodological Issues and Suggestions for Future Research

Interpretation of the results presented in this review is made difficult by a number of conceptual and methodological issues in the research area. One of the most prominent issues to arise is the lack of consistency in the use of terminology relating to dispositional mindfulness. Rau and Williams (2016) touched upon the suggestion that research risks portraying all forms of mindfulness as the same construct. In line with this, throughout the process of conducting this systematic review, it was clear that mindfulness is often used an umbrella term to encapsulate both dispositional mindfulness and mindfulness therapy, irrespective of the fact that these are vastly different constructs. In the future, authors should aim to clearly state the aspect of mindfulness they are exploring. This will help to promote transparency within the literature and foster a clearer distinction between different types of mindfulness.

There are also issues relating to the DM measures used. Grossman (2011) questioned the validity of DM measures, expressing uncertainty over whether they actually measure mindfulness or some other construct. Further, it has been noted that there is no agreed ‘gold standard’ for mindfulness instruments and there is ‘a lack of available external referents for determining construct validity’ and a ‘convergent validity among different mindfulness scales’ (Grossman 2011, p. 1034). This review found that DM is most commonly assessed as a one-dimensional construct by the MAAS (Brown and Ryan 2003). This has been discouraged, with some arguing that tools such as the MAAS are oversimplified (Grossman 2011). Instead, it has been argued that DM should be assessed as a multi-faceted construct (Rau and Williams 2016), e.g. by using the FFMQ, which was found to be the second most commonly used measure in this review. It is important to assess the links between facets of DM and psychological outcome variables as different facets may have different effects on health. This was found to be the case in research using the FFMQ by Adams et al. (2012). They found that DM facets ‘describing’ and ‘non-judging’ predicted lower eating pathology and body dissatisfaction, whilst ‘observe’ predicted higher anorexic symptoms. Further exploration between specific DM facets and psychological health is needed as it will help to aid the development of effective patient-centred interventions. In the future, researchers should aim to use multi-faceted DM measures and avoid adding up facet scores to form a total score, as this effectively makes an average of correlated and uncorrelated facets, forming an inaccurate picture of the relationship between DM and the outcome variable (Baer et al. 2006).

Despite promoting the use of multi-faceted DM measures such as the FFMQ, it has been argued that the factor structure of this measure may need to be re-evaluated first (Baer et al. 2006; Petrocchi and Ottaviani 2016). Studies show that the ‘observe’ facet of this scale has low test-retest reliability and has demonstrated non-significant or negative correlations with the other four facets of DM (Baer et al. 2004). Dropping this facet may therefore be advisable, as it currently negatively affects the validity of the measure (Siegling and Petrides 2016). Future research needs to look to improve the reliability and validity of tools to measure DM and develop methodology to reliably distinguish between state and trait measures and use it to validate existing psychometric instruments.

This review has identified that the research in this area uses predominantly quantitative (questionnaire-based) methodologies (the number of qualitative papers excluded from the review were few). Additionally, by following an established procedure to narrow down the search engine results, four key terms were used through which to explore the link between DM and psychological health: moderate, mediate, predict and correlate. This would have fostered the finding of more quantitative studies. The frequent use of self-report inventories expose studies to significant response bias and allow only a certain depth of findings (Kabat-Zinn et al. 1985). Future research may benefit the field by employing qualitative methods, which could shed more light on some of the existing findings by a more in-depth investigation of the phenomena. More longitudinal studies, such as that by Petrocchi and Ottaviani (2016), can also help to explore the effects of DM over time. Additionally, this review has identified that often ordinal data is used with parametric tests, violating the assumptions of analysis. Future research should overcome this by using Rasch analysis to transform ordinal data into interval data to improve precision of measurement and reliability of analysis (Medvedev et al. 2016).

Lastly, the research outlined is limited due to predominantly being conducted with student populations of mainly white Caucasian individuals. More research using more representative samples would enhance external validity of the results. In particular, as there is a large literature focusing on the positive effects of DM on stress reactivity and recovery, researchers should strive to explore this in populations that are exposed to more stressful situations and are more vulnerable to the ill effects of stress, for example marginalised groups such as ethnic minorities and disabled individuals (Thoits 2010). This will ensure that results can be applied to those who may need it most. Additionally, although there has been some research in this area demonstrating the psychological benefits of DM in older adults (Mahoney et al. 2015; Paolini et al. 2012; Prakash et al. 2015), less has been carried out with children and younger age groups. It is likely that DM will exhibit the same benefits in younger adults and children, and if this is found to be the case, there is argument to target schools to boost DM in school-aged children. It is possible that this might enhance emotion regulation and decrease maladaptive thinking styles among children.

### Limitations

This review included only published articles in English. Papers published in other languages may give further clarification of the links between DM and psychological health; this may be particularly valuable because non-English articles can shed some light on this phenomenon in other cultures. Moreover, the search terms were searched in the titles and abstracts of articles, which may have left out some research whose focus was different but contributed to DM and psychological health in some capacity. The review is strengthened, however, by including papers from a wide range of countries, suggesting that the findings have high cross-cultural external validity.

In conclusion, this review has demonstrated that DM is positively related to psychological health on a range of outcome measures. DM appears to be inversely associated with a variety of psychopathological symptoms and studies suggest that the underlying cognitive processes may be a mediating factor in this relationship. DM appears to buffer against the propensity to engage in negative thinking patterns, which is a risk factor for depressive symptoms. Emotional factors such as well-being and emotional regulation also appear to be benefited by DM. These findings should be used within a proactive approach to boost DM to promote well-being, resilience and self-management of psychological health within the general population. This review shows that there are several avenues for future research and has outlined conceptual and methodological limitations within the field such as issues with DM measures, unsuitability of ordinal data for parametric tests, sample selection and the use of inconsistent terminology. These issues should be overcome in future studies to progress this area of research.
